# Treatment with Calcium Ionophore Improves The Results in Patients with Previous Unsuccessful Attempts at The Fertilization: A Cohort Study

**DOI:** 10.22074/IJFS.2021.136168.1013

**Published:** 2021-10-16

**Authors:** Alberto Tejera, Lucia Alegre Ferri, Pilar Gamiz Izquierdo, Diana Beltrán Torregrosa, Jose Alejandro Remohí, Marcos Meseguer Escrivá

**Affiliations:** Instituto Valenciano de Infertilidad, Universidad de Valencia, Valencia, Spain

**Keywords:** Calcium Ionophore, Intracytoplasmic Sperm Injection, Male Factor

## Abstract

**Background:**

The objective of this study is to evaluate artificial oocyte activation (AOA) with calcium ionophore (CaI) in a subsequent attempt at fertilisation in patients after extremely low or failed fertilisation. We assessed improvements in fertilisation, implantation and pregnancy rates as well as cancellation rates in these patients. Finally, was evaluated the result testing in addition to delivery rate and obstetric outcomes in children born after AOA.

**Materials and Methods:**

This was a retrospective observational study conducted in an IVF laboratory of an IVI clinic (IVIRMA Valencia, Spain). One group (509 mature oocytes from 66 patients) received a first intracytoplasmic sperm injection (ICSI) without AOA, which resulted in either a failed fertilisation or very low values (<30%). This group was compared with a second group (616 mature oocytes from the same 66 patients) that used AOA. Outcome was compared by McNemar’s test and the dependent t tests.

**Results:**

AOA plus CaI resulted in enhanced fertilisation (51 vs. 13.1%), ongoing pregnancy (47 vs. 21.7%), and implantation (31.1 vs. 13.1%) rates, and less chances for cancelling the cycle (22.7 vs. 69.3%). There were no observed adverse effects in obstetric and perinatal outcomes after the use of AOA.

**Conclusion:**

Our findings support the use of AOA for a given population of patients where fertilisation was affected during previous attempts. After AOA, we observed a significant increase in reproductive success due to the increased number of embryos available for embryo selection and, therefore, enhanced chances for success. The use of this artificial technique is comforting after checking non-existence of detrimental effects on the offspring.

## Introduction

Implementation of intracytoplasmic sperm injection (ICSI) in our laboratories enabled us to overcome most problems related to male factor infertility that were attributed to low counts and diminished sperm motility. However, there are a low percentage of cycles where conventional ICSI does not work as expected; consequently, around 1-3% of cycles have complete fertilisation failure or a low fertilisation rate compared to the average of 70-80 % (1).

Naturally, oocyte activation occurs after sperm-oocyte fusion, which is induced by a series of intracellular calcium oscillations that are generated and released from endoplasmic reticulum stores after the sperm enters the cytoplasm. These oscillations pursue until pronuclear (PN) formation (2-4), and then they stop. Most of these calcium oscillations have been studied in mammals and identified as phospholipase PLC zeta (PLCƺ), a protein located in the perinuclear theca of spermatozoa. Under usual conditions, this factor enters the oocyte’s ooplasm and causes release of calcium (5) followed by activation of the egg and resumption of the cell cycle. It is proposed that many fertilisation failures in infertile men are due to a lack of oocyte activation because of deficient intracellular calcium release, especially the oscillation cascade (6, 7), with the exception of those cases that have incomplete cytoplasm oocyte maturation where the response to this sperm factor is inhibited (8).

One well-known option for rescuing these unsuccessful cycles consists of increasing the amount of calcium into the ooplasm through the use of ICSI followed by artificial oocyte activation (AOA), which is called ICSI-AOA - a modified technique of conventional ICSI (9). ICSI-AOA is induced by the use of chemical substances, calcimycin (A23187) and ionomycin, which are the most widely used because of excellent results (10-12).

The importance of calcium oscillation during the activation time becomes evident on successive pre- and post-implantation events - embryonic development and pregnancy outcomes (2). In contrast, AOA, by the use of electrical or other chemical methods in humans, causes an only one abnormal calcium gain without the posterior needed frequency of calcium oscillations.

Montag et al. (13) published a retrospective study with fertilisation rates close to 50% after the use of calcimycin (A23187) in previous cases with fertilisation problems. Similarly, patients with either minimal or nonexistent fertilisation values (<30 %) would benefit from the use of AOA with CaI (11, 13). Recently, another author (14) published a randomized clinical trial that found benefits in subsequent treatments with the use of AOA in couples with male factor and unsuccessful conventional ICSI in previous cycles. The published benefits and evidence for the use of AOA make this strategy a potential option for certain patients. 

We evaluated the effect of CaI on patients who had previous failed attempts with conventional ICSI and, as a consequence, in the following cycle they were treated with ICSI-AOA. Analyses of the perinatal and obstetric effects during and immediately following birth in children derived from oocytes treated with AOA were also performed.

## Materials and Methods

The Instituto Valenciano de Infertilidad (IVI Valencia) performed this retrospective study over the last four years. This study was approved by the Institutional Review Board, Ethical Committee of Clinical Research IVI Valencia (ref. 1506-VLC-045-MM), which regulates and approves database analysis and clinical IVF procedures for research at IVI Valencia. It also complies with the Spanish law governing assisted reproductive technologies (14/2006). 

We included all patients registered in our electronic medical records system. There were 66 patients included in this study with a total number of 163 cycles. The first group consisted of 75 cycles, 509 oocytes and 41 embryos. We included patients with previous conventional ICSI treatments (without AOA) that resulted in compromised fertilisation (fertilisation failure or fertilisation below 30%). These patients presented with good response and oocyte quality (no significant morphological abnormalities) and non-severe male factor. Subsequently, they underwent conventional ICSI. The second group (with AOA) consisted of the same patients (n=66) who did not succeed in the first attempt with conventional ICSI treatment. After an unexpected low (<30%) or absent fertilisation rate, AOA treatment was indicated for the second attempt to improve the fertilisation outcome. This second group generated 88 cycles, 616 oocytes and 104 embryos. Both groups 1 and 2 had fresh and frozen embryo transfers.

The primary outcome of this study was ongoing pregnancy rate and the secondary outcomes included fertilisation, implantation, miscarriage, and live birth rates, usable embryos and percentage of cycles with surplus embryos in each group. Patients older than 39 years of age and those subjected to egg donation were excluded from the study.

### Ovarian stimulation, oocyte pick up and denudation

The same stimulation protocol was used for both groups because they were the same patients. Gonadotrophin-releasing hormone (GnRH-a) antagonist protocols were employed from day 21 of the cycle by subcutaneous administration for controlled ovarian hyperstimulation (15, 16). Briefly, once the ovarian quiescence was confirmed, a combination of recombinant follicle stimulating hormone (rFSH, Gonal-F, Merck Serono) and human menopausal gonadotrophin (hMG, Menogon, Ferring) were administered at a 2:1 ratio, and the dose was adjusted according to the response. After three or more follicles reached 17 mm in diameter, final maturation was induced by administration of 10 000 IU human chorionic gonadotropin (hCG) and, after 36 hours, follicles were aspirated and washed and placed into Global HEPES medium (LifeGlobal) at 37°C by using a tube warmer. The encountered eggs were continuously kept in an incubator for 3-4 hours before they were denudated.

Between three and four hours after oocyte harvesting, denudation was conducted by mechanical pipetting in 1:1 Global hyaluronidase (80 IU/mL; LifeGlobal) and Global w/HEPES by passing the oocytes through increasingly smaller denuding pipettes from 275 to 150 µm). Once granulose cells were removed oocyte maturation was confirmed under a microscope, and we selected the metaphase II (MII) oocytes for microinjection

### Intracytoplasmic sperm injection-artificial oocyte activation and conventional ICSI procedures

Semen samples were collected by masturbation into non-toxic sterile plastic jars after 3-5 days of sexual abstinence. The samples were allowed to liquefy for 30 minutes at room temperature (22°C) before they were evaluated according to WHO criteria.

For the density gradient centrifugation (DGC) procedure, ALLGrad® (LifeGlobal®, Guelph, Canada) was diluted in medium for Global Fertilisation® (LifeGlobal®) to obtain dilutions of 45% and 90%. Two gradient columns were prepared in Falcon® tubes by gently layering 1 ml of each solution, starting with the 90% fraction at the bottom. One ml of the semen sample was centrifuged for 10 minutes at 350 g with Global Fertilisation® before it was stratified on top of the discontinuous gradient columns and centrifuged for 18 minutes at 300 g. After centrifugation, the pellet was collected and centrifuged twice at 350 g for 5 minutes. After DGC, a second evaluation of sperm parameters was carried out and the prepared samples were used for microinjection of the oocytes.

Approximately four hours after retrieval and two hours after denudation, ICSI was performed under a microscope at x40 x10 (magnifications) in both groups.

As mentioned above, the ICSI procedure for the study patients was somewhat different from the standard procedure. This combined method (ICSI-AOA) was performed as described. Briefly, a spermatozoon was injected into the oocyte in a conventional way. Consecutively, the oocytes were exposed to a pre-equilibrated calcium ionophore (CaI) (GM508 CultActive, GYNEMED) for 15 minutes.

The solution consisted of NaCl, KCl, KH_2_ PO4, MgSO_4_ , 7H_2_ O, NaHCO_3_ , CaCl_2_ .2H_2_ O, D(+)-glucose anhydrous, Na-lactate, Na-pyruvate, EDTA, alanyl-glutamine, water, non-essential and essential amino acids, DMSO, and Ca+2 ionophore A23187. This medium (GM508 CultActive) is a bicarbonate-buffered reagent designed for oocytes of patients with failed fertilisation after previous ICSI cycles. The spermatozoa were selected according to morphological criteria without selecting borderline or abnormal sperm whenever possible, and then immobilized in the same way as conventional ICSI (fracturing the flagellum with the ICSI needle). 

Immediately after the injection, the oocytes were incubated for 15 minutes in pre-equilibrated (4 hours) Ca^+2^ - ionophore GM508 at 37ºC, 6% CO_2_ and 20% O_2_ atmosphere. Next, the oocytes were washed in clean Global (LifeGlobal) media drops and placed in new culture dishes (Vitrolife) that contained Global media pre-incubated overnight at 37ºC, 6% CO_2_ and 20% O_2_ atmosphere until embryo transfer or vitrification were conducted.

In the control group, ICSI was performed as usual, without any contact between sperm or oocytes with the CaI solutions, and according to the standard process. The microinjected oocytes were cultivated in the same culture conditions as the study group (Global media dishes at 37ºC, 6% CO_2_ and 20% O_2_ atmosphere).

### Fertilisation and embryo assessment

Fertilisation assessment and embryo classification were conducted on days 1, 3 and 5 of culture according to established methods (17, 18). Embryo selection for transfer or cryopreservation was performed on day 3 or day 5 depending on the embryo’s features and quality.

Depending on the patient’s history, single embryo transfer (SET) or double embryo transfer (DET) was offered with a maximum of two embryos transferred and, rarely, three embryos when the previous history promoted to transfer them. Wherever possible, elective SET was performed. 

Supernumerary embryos were vitrified for further frozen embryo transfers (17, 19).

### Assessment of clinical outcomes

A serum pregnancy test was conducted for β-hCG levels at 13 days post-transfer. Clinical pregnancy was defined as the presence of at least one intrauterine gestational sac with a foetal heartbeat detected by ultrasound examination, up to 12 weeks after the pregnancy test. Both live births and neonatal outcomes were reported.

### Fresh and deferred cycles

In the standard group, 41 embryos were transferred as follows: 18 fresh cycles with 28 cleavages and 3 blastocyst embryos; 4 frozen cycles with 7 cleavages and 1 blastocyst embryo; one mixed cycle with two cleavage embryos (1 frozen embryo joined to 1 fresh embryo); and 52 total cancelled cycles. Of these, 48 fresh cycles were cancelled because neither the embryo nor the zygote were obtained after injection. In addition, four cases (the embryos were vitrified for deferred transfer) were cancelled after warming due to a lack of embryos for transfer or no surviving embryos.

In the AOA group, 104 total embryos were transferred as follows. There were 52 embryos transferred in 31 fresh cycles: 21 blastocyst embryos in 14 cycles and 31 cleavage embryos in 17 cycles. There were 52 frozen embryos transferred in 37 deferred cycles: 44 blastocyst embryos in 31 cycles and 8 cleavage embryos in 6 cycles. There were 20 cycles terminated: 15 fresh cycles were cancelled due to either poor embryo quality or no fertilisation was achieved and five thawed cycles were cancelled after verification that there were no surviving embryos. 

Both 37 cycles from AOA and 4 cycles from the standard group were treated as deferred embryo transfers because the endometrium was not receptive for embryo transfer as well as hydrometra or ovarian hyperstimulation syndrome (OHSS) were observed. In these cases, the embryos were frozen for the next endometrium preparation.

In both the standard and AOA group, the embryos were replaced either at the cleavage or blastocyst stages according to medical indication, previous history and after counselling by the specialist team.

### Statistical analysis

The total number of retrieved oocytes was 1348. Of these, we obtained 1125 mature oocytes (83.5%) that were divided into two groups; 616 treated with AOA (study group), and 509 treated by conventional ICSI (control group). Patients were compared (repeated) with or without CaI, and then related to the comparisons. We used McNemar’s test for categorical variables while the t test was used for dependent samples, differing between variables. P<0.05 was considered statistically significant. The analysis included fertilization, pregnancy, ongoing pregnancy, and implantation rates as well as cancellation and delivery rates to determine the effectiveness of the AOA procedure. All analyses were performed using the Statistical Package for the Social Sciences 24 (SPSS, Chicago, IL, USA). Finally, multivariable logistic regression that included the generalised estimating equations (GEE) procedure to overcome the comparison within the same patients was conducted to assess the effect of AOA on ongoing pregnancy per cycle as well as per transfer.

## Results

A total of 1125 microinjected oocytes were analysed in terms of fertilisation rate (normal or abnormal), degeneration rate, ongoing and implantation rates, and cancellation rate as well as embryo development. A comparison and analysis of the data in general terms showed that the AOA group (51%) had a higher fertilisation rate than the control group (13.1%, P<0.001). The AOA group also had a better ongoing pregnancy rate (47 vs. 21.7%, P<0.05) and implantation rate (31.1 vs. 13.1%) compared to the control group; however, there were no significant differences. Lower chances to cancel the cycle (22.7 vs. 69.3%, P<0.001) were found by applying AOA with CaI. The average embryo transfer was similar: 1.72 for the standard group and 1.66 for the AOA group. 

As reflected in Table 1, the average sperm count, maternal age and number of matured oocytes, as well as the percentage of blastocyst transferred in each group and viable embryos (frozen or transferred) between the two groups were analysed by observing differences in the percentages provided by each group. In the AOA group, 66% of the cases were transferred during the blastocyst stage whereas in the conventional group, only 17.4% of the cases (P<0.01). The AOA group reached 33.1% of viable embryos and the control group reached the double 61.2% (P<0.01).

**Table 1:** Sperm, oocyte and embryo data


Type of technique	ConventionalICSI	AOA	P value

Paternal age (Y)	35.8(35.1-36.5)	36.1(35.4-36.7)	NS
Sperm count (10^6^/ml)	18.2(0.01-89.0)	24.5(0.01-170.0)	NS
Sperm count (mill/ml after DGC)	3.93(0.01-21.0)	4.01(0.01-25.0)	NS
Maternal age (Y)	35.1	35.6	NS
Number of MII per cycle	6.73(2-16)	7.68(1-20)	NS
Number of embryos transferred	1.72(1-3)	1.66(1-3)	NS
Average embryos frozen	0.21(0-2)	1.35(0-8)	<0.001
Blastocyst transfer	17.4% (4/23)	66% (45/68)	<0.01
Good quality blastocysts	17.6%	19.9%	NS
% of usable embryos (transferred or frozen)	61.2% (41/67)	33.1% (31/104)	<0.01


Data are presented as average with a confidence interval (CI) of 95% between brackets. Average of sperm count in fresh and after DGC, mean age, average number of oocytes, and number of embryos transferred in each group as well as percentage achieved in terms of frozen or viable embryos according to the applied different technique. ICSI; Intracytoplasmic sperm injection, AOA; Artificial oocyte activation, DGC; Density gradient centrifugation, NS; Non-significant, and MII; Metaphase II.

Table 2 shows the variables related to both normal (two polar body and two pronuclei) and abnormal fertilization (one pronuclei or more than two pronuclei), and the degeneration rate after microinjection according to the method used. 

**Table 2:** Maturation and fertilisation rates


Type of technique	Conventional ICSI	AOA	P value

Oocyte numbers	605	743	-----
Matured oocytes (1125)	509	616	-----
2PN/MII rate	67/509 (13.1%)	314/616 (51.0%)	<0.001
Abnormal fertilization rate	40/509 (7.8%)	27/616 (4.4%)	NS
Degeneration rate	31/509 (6.1%)	52/616 (8.4%)	NS
% non-fertilized oocytes	371/509 (73%)	223/616 (36.2%)	<0.001


Data are presented as average with a confidence interval (CI) of 95% between brackets. Normal and abnormal fertilisation rate and non-fertilisation rate as well as degeneration rate after microinjection according to both techniques used in this study. ICSI; Intracytoplasmic sperm injection, AOA; Artificial oocyte activation, 2PN; 2 pronuclear, MII; Metaphase II, NS; Non-significant, Normal fertilization or 2PN; Zygotes with two polar bodies and two pronuclei, and Abnormal fertilization; Zygotes that are unipronuclear or with more than two pronuclei, in addition to those without extrusion of the second polar body and without pronuclei due to lack of oocyte activation.

Table 3 shows the other analysed variables regarding outcome of the cycle, cancellation rate and development cycle rates. Group 1 consisted of 18 fresh cycles, 5 frozen cycles and 52 cancelled cycles due to poor embryo quality, no zygotes obtained or no embryos that survived post-thawing.

**Table 3:** Logistic regression analysis by cycle and transfer


Type of technique	Conventional ICSI	AOA	P value

Cycles (163)	75	88	---------
Cancellations	52/75 (69.3%)	20/88 (22.7%)	<0.001
Transferred-fresh cycles	18/75 (24%)	31/88 (35.2%)	NS
Transferred-frozen cycles	5/75 (6.6%)	37/88 (42%)	<0.05
Total cycles with embryo transfer	23	68	------
Ongoing pregnancy	5/23 (21.7%)	32/68 (47%)	<0.05
Ongoing pregnancy rate (fresh cycle)	2/18 (11.1%)	13/31 (42%)	<0.05
Ongoing pregnancy rate (frozen cycle)	3/5(60%)	19/37 (51.35%)	NS
Transferred embryos	41	104	------
Implantation rate (100%)	5/41 (12.2%)	32/104 (30.8%)	NS
Miscarriage rate	3/5(60%)	3/32 (9.3%)	NS
Babies born	3	22	-----
% of cycles with surplus embryos	1/75 (1.3%)	15/88 (17%)	<0.05


Data are presented as average with a confidence interval (CI) of 95% between brackets. Cancellation and development cycle rates were compared between the two groups, as well as ongoing pregnancy and implantation rates that resulted from embryos obtained by either technique. ICSI; Intracytoplasmic sperm injection, AOA; Artificial oocyte activation, and NS; Non-significant.

Group 2 consisted of 31 fresh cycles and 37 frozen cycles. Unfortunately, 15 fresh cycles were cancelled due to the lack of an embryo for transfer and 5 frozen cycles were cancelled because embryos did not survive or inadequate embryo development post-warming. The overall ongoing pregnancy rate was higher in the AOA group (47%) compared to the conventional group (21.7%, P<0.001). When separated according to type of cycle (fresh or frozen), we noted that the ongoing pregnancy rate was higher in the AOA group (42%) compared to the conventional group (11.1%) in the fresh cycles. However, in the frozen cycle, the results were quite similar (60% in the conventional group and 51.3% in the study group, P<0.05). The implantation rate was higher for the AOA group (31.1%) compared to the conventional group (13.1%). In terms of cycles with frozen surplus embryos, the standard group achieved one cycle with one frozen embryo (1/75, 1.3%), whereas there were 15 cases with surplus frozen embryos (15/88, 17%) in the AOA group (P<0.05).

The number of babies born according to technique and the neonatal and obstetric data are shown in Table 4. In the control group, two pregnancies resulted from fresh embryo transfer and three pregnancies occurred after frozen embryos were transferred. Although there were 5 successful cases out 15, 3 ended in clinical pregnancy lost. As a result, three babies were born (1 from a singleton delivery and 2 from twins) via vaginal delivery. Out of 21 ongoing pregnancies achieved by AOA, 13 newborns originated from 13 frozen embryo transfers (10 blastocyst stage transfers and 3 cleavage stage transfers). Eight newborns resulted from 7 fresh embryo transfers (4 blastocyst stage transfers and 3 cleavage stage transfers). As a result, 20 of them gave rise to 21 babies born (one twin pregnancy) and one was lost in early pregnancy. Of these, 9 were born by caesarean section and 12 delivered vaginally. 

Perinatal and obstetric outcomes were studied after the births of the babies from both techniques (Table 4). There were no minor or major adverse effects noted in the offspring. It was remarkable that there were low birth weight babies in the conventional group; however, the sample size was limited and might not be considered (three babies). 

Although no differences were observed between ICSI and ICSI-AOA patients in terms of the day of transfer and maternal age, a further analysis was performed by multivariable logistic regression in which those variables were included as potential bias factors. The study was performed to weigh the effect of AOA on ongoing pregnancy per cycle as well as per transfer. As demonstrated, the application of AOA in our patients increased by more than four times odds ratio (OR=4.57) per cycle, but not per transfer. When embryos were available for transfer, AOA did not increase the chances of a viable pregnancy (Table 5)

**Table 4:** Neonatal data


Newborns data	Conventional ICSI	ICSI-AOA

Live birth rate	7.3% (3/41)	20.2% (21/104)
Weeks at delivery	37.4 (37.1-37.6)	39.2 (36.6-41.5)
Preterm births(<37 weeks)	0 (0%)	1 (5%)
Very preterm births (<34 weeks)	0 (0%)	0 (0%)
Caesarean section	0 (0%)	8 (38.1%)
Vaginal delivery	3 (100%)	12 (60.0%)
Neonatal outcome	N=3	N=21
Gender		
Female	1 (33%)	8 (38%)
Male	2 (66%)	13 (62%)
Birth weight (g)	2253 (2115-2365)	3361 (2500-4300)
LBW (<2500 g)	3 (100%)	0 (0%)
Neonatal height (cm)	45.3 (44-46)	49.8 (45-53)
Apgar <7 at 5 minutes	0 (0%)	0 (0%)
Apgar score at 1 minute	9 (9-9)	9 (9-9)
Apgar score at 5 minutes	9 (9-9)	9.9 (9-10)
Malformations	0	0
Major malformations	0 (0%)	0
Minor malformations	0 (0%)	0 (0%)
Neonatal intensive care	0	2
Perinatal mortality	0 (0%)	0 (0%)


Data are presented as average with a confidence interval (CI) of 95% between brackets. Neonatal data and birth defects were retrieved for all children born through both techniques as well as preterm birth data, weeks of delivery and type of birth. The number of cases and/or range (between brackets) is included. ICSI; Intracytoplasmic sperm injection, AOA; Artificial oocyte activation, and LBW; Low birth weight.

**Table 5:** Sperm, oocyte and embryo data


Model effect	Value	OR	P value
Ongoing pregnancy rate per cycle			

ICSI	AOA versus conventional	4.57 (1.47-13.97)	0.008
Day of transfer	Day 3 versus day 5	0.93 (0.84-1.04)	NS
Maternal age	Years	0.80 (0.50-1.26)	NS
** Ongoing pregnancy rate per transfer**			
ICSI	AOA versus conventional	0.964 (0.23-4.06)	NS
Day of transfer	Day 5 versus day 3	0.85 (0.51-1.41)	NS
Maternal age	Years	0.73 (0.10-4.91)	NS


Logistic regression analysis of ongoing pregnancy after week 12 as affected by ICSI-AOA. The effect was considered by cycle and by transfer. As co-variables we included the day of transfer (day 5 vs. day 3) and age of the patient (years) because co-variables were included. OR is shown with 95% confidence intervals in brackets. OR; Odds ratio, ICSI; Intracytoplasmic sperm injection, and AOA; Artificial oocyte activation.

## Discussion

The ICSI procedure must be performed by experienced personnel because an improperly performed ICSI technique in any* in vitro* fertilization (IVF) laboratory would imply a decrease in fertilisation rate. Occasionally, there are very low number of collected oocytes that might not yield embryos for transfer. In any case, both of the above mentioned options are not responsible of this issue at hand. That is particularly the case where the ICSI is not successful, as we expected, even when the technique is carried out by senior embryologists and uses a good number of oocytes. Although the majority of technologies employed in assisted reproductive techniques have largely resolved infertility problems, there are still unsuccessful ICSI cycles due to improper oocyte activation. Oocyte activation did not occur in approximately 70% of oocytes unfertilised after sperm injection. In most of these cases, activation deficiency was due to PLCƺ impairment (aberrant function), as shown by the reduced levels or absence of its expression (20). Nevertheless, some studies have reported that, punctually PLCƺ protein seems to be unstable and could not be as effective as expected; therefore, the application of AOA is a requirement for cases with previous unsuccessful fertilisation attempts in order to achieve pregnancy (21). Our current method, which we reported as modified ICSI, used an AOA methodology with proven improvement in terms of fertilisation, embryo development and success rates (22).

The AOA technique has not resulted in any minor or major problems in offspring that resulted from this burgeoning technique (21). In the last decade, AOA treatment has been successfully tested in IVF laboratories in cases of highly altered sperm quality like globozoospermia or teratozoospermia (21, 23, 24).

However, the benefit has also found for patients with normozoospermia (as in our case) where were not obtained embryos for transfer in the first attempt, but after AOA implementation were achieved better results and it was reported in consequence (25). 

Tavalaee et al. (25) found a benefit with the use of AOA for cases with extreme changes in semen (globozoospermic) where inappropriate levels of PLCƺ was the reason for oocyte inactivation and subsequent fertilisation failure. Their results showed that fertilisation rates after AOA in globozoospermic patients matched our results (53.14 vs. 51); however, they used globozoospermic patients as a study group whereas our study population consisted of patients with normal semen (apparently no impaired), which probably masked compromised levels of PLCƺ.

Other researchers performed a more detailed assessment that checked the levels of PLCƺ in infertile men who had histories of failed oocyte activation (26). They found a decreased percentage of relative expression of this protein in infertile men as well as globozoospermic men compared to fertile men or patients with high fertilisation rates.

In our study, we observed an improvement in cases where the oocyte activation was harmed (most probably due to sperm, despite normal morphology and count parameters). Although we did not study the phospholipase levels in the oocytes, the data caused us to ponder PLCƺ dysfunction in the study group and, therefore, this dysfunction might be liable for this fertilisation failure. Although the proportion of this alteration is extremely rate (1% of men), it was published by some authors (27). By taking into consideration the number of treatments performed in our centre over four years (approximately 10000 retrievals, excluding severe male factor cases), this very low rate of approximately 80 cycles matches the failed cases without AOA due to hidden male factor.

Therefore, we can recover egg activation, resume the first cell cycle and consequently improve oocyte fertilisation, and obtain more embryos after treatment, which would increase the chances for success. The most common CaI suppliers are ionomycin and calcimycin (1, 3, 28, 29), both for injection and incubation for 10 minutes or for injecting oocytes according to conventional ICSI and incubating them for 15 minutes in a CaI solution. Both options provoke the flow inlet of extracellular calcium. In our study, we preferred to use the second option (conventional injection and culture) to guarantee the success of the procedure without potential injury after injection.

This increase of intracellular calcium would re-establish normality and overcome the first calcium wave that should be caused by PLCƺ. This calcium release caused by the effect of A23187 can mimic the repeated natural oscillations by calcium that are necessary to complete the cell cycle (30).

Another aspect to consider is the asynchrony between cytoplasmic and nuclear maturation. It has been proposed that oocytes with incomplete cytoplasmic maturity lead to poor clinical outcome, because of the association with a lower than expected proportion of MII oocytes (31). In our study, the percentage of matured oocytes (MII) obtained in the AOA group was similar to the control group (82.9 vs. 84.1%), and did not affect the fertilisation rate by the poor cytoplasmic maturity.

The implementation of AOA in the laboratory daily routine has allowed us to obtain a double benefit: on the one side, the pregnancy chances of the couple were increased due to more zygotes and, consequently, more embryos and, on the other side, we allowed the use of homologous gametes (bypassing unneeded sperm donation), especially when the couple wished to repeat the treatment with guarantees after a fertilisation failure.

Different authors reported high fertilisation levels (74%) and acceptable pregnancy rate limits (33%) after using AOA in couples with previous failed fertilisation. Regarding the fertilisation rate, our values were lower (74 vs. 51.3%); however, our pregnancy rate was superior to those found by Heindryckx et al. (11) (33 vs. 45.7%). Our results are in line with those found by Ebner et al. (30), who performed a study similar to ours and reported the following benefits after AOA in previous untreated cycles: fertilisation rates (56.9 vs. 51.3%), implantation rates (33.3 vs. 32.6%), and pregnancy rates (29.7 vs. 45.7%). Even when taking into account the existing limitation of frozen embryos, the live birth rate between both groups significantly increased (10.7 vs. 29.4%) and 23 babies were born after both techniques: 16 singleton and 2 twin pregnancies, which resulted in 20 healthy children after AOA; one twin pregnancy and one singleton gave rise to three babies in the conventional ICSI group. There were no observed malformations.

An interesting paper from Belgium (32) described the neonatal and developmental outcome in 21 babies born following AOA. Their conclusions were reassuring, since the children born following AOA had normal neonatal, developmental and behaviour outcomes and there were no serious unfavourable effects observed. Another paper reported 22 babies born after different types of AOA where they studied 10 babies born after the use of CaI A23187 and 12 following SrCl_2_ ; the infants had similar health and growth. Other authors assessed the weights and heights of 21 babies (up to six years) born after AOA, and found physical growth within the 10-90% percentile (32).

We obtained frozen surplus embryos that resulted from AOA (17 patients obtained extra embryos and 10 patients stored frozen embryos for future use), while in the control group, there was only one frozen embryo; thus, these results would be more promising. Thus far, the published studies found the same results as the current study results (no deleterious effects in the children born) with a similar number of babies born. Secondly, AOA is a safe option for couples, especially after failed conventional ICSI, as long as there is no oocyte factor hidden like immature oocyte factor (33, 34).

The difference in blastocyst transfer achieved with the two groups (17.4 vs. 66%) was remarkable. This difference was probably due to the lower number of embryos obtained by the conventional group, which promoted us to advance the embryo transfer on day 3. In this context, it should be noted that the higher proportion of usable embryos in favour of the conventional group demonstrated that once the embryos were obtained, the chances of pregnancy were the same. 

## Conclusion

A limitation of this study was the absence of PLCƺ analysis in the study group. A more detailed study should analyse PLCƺ levels in patients with compromised fertilisation to confirm the current study findings.

Both ongoing pregnancy and implantation rates, the number of embryos frozen per cycle and the percentage of cycles with surplus embryos were improved using AOA. Therefore, based on these results, we encourage the scientific community to develop more studies to confirm the effectiveness of AOA. The existing evidence recommends us to offer this type of treatment to patients with previous poor fertilisation procedures where we know or we are almost certain that the previous fertilisation failure was attributed to the sperm.
